# Comparison of different concentrations atropine in controlling children and adolescent myopia: an umbrella review of systematic reviews and meta-analyses

**DOI:** 10.3389/fopht.2024.1447558

**Published:** 2024-10-23

**Authors:** Baizhou Chen, Yao Ni, Jinghan Chen, Shuwen Xing, Zhaotian Zhang

**Affiliations:** ^1^ State Key Laboratory of Ophthalmology, Zhongshan Ophthalmic Center, Guangdong Provincial Key Laboratory of Ophthalmology and Vision Science, Guangdong Provincial Clinical Research Center for Ocular Diseases, Sun Yat-sen University, Guangzhou, Guangdong, China; ^2^ Department of Visual Science, Guangzhou Xinhua University, Guangzhou, China

**Keywords:** myopia, atropine, concentration, children, axial length, spherical equivalent refraction (SER)

## Abstract

**Purpose:**

To evaluate the myopia control effect of different concentrations atropine in children and adolescent.

**Methods:**

Meta-analyses and systematic reviews available in the Pubmed, Embase, and Cochrane Library databases from the databases’ inception to August 2023 were searched to evaluate the efficacy and tolerability of different concentrations’ atropine in controlling myopia progression. Overall effects were performed using random-effects model. AMSTAR 2 tool was used to assess the quality of included studies. Prespecified outcomes were weight mean difference (WMD) with 95% credible interval (95% CI) of annual spherical equivalent refraction (SER) changes and annual axial length (AL) changes.

**Results:**

19 systematic reviews/meta-analyses of different atropine concentrations were included in the analysis. 14 studies reported SER changes, and 17 reported AL changes. In terms of the studies’ overall methodological quality level (measured using AMSTAR 2), 1 study was rated high, 7 moderate, 7 low, and 4 critically low. The 0.01% atropine was found to have statistically significance (annual SER change WMD 0.27 [95% CI 0.21 - 0.34] D/year; annual AL change WMD -0.09 [95% CI -0.1 to -0.07]) mm/year), 0.05% atropine was preferred considering efficacy and tolerability (annual SER change WMD 0.54 [95% CI 0.49 - 0.58] D/year; annual AL change WMD -0.21 [95% CI -0.12 to -0.02]) mm/year).

**Conclusions:**

Different atropine concentrations alleviated children and adolescent myopia progression. However, higher-quality evidence and further investigation are needed to clarify the dose–response relationship, and practical guidelines must be developed to determine myopia control efficacy.

## 1 Introduction

Myopia has increasing prevalence and has raised wide public concern in the last few decades, especially in Asia (1–3). It is estimated that 49.8% of the world population will suffer from myopia by 2050, and that 9.8% will have high myopia (4). School-age children and adolescents are the main group suffering from myopia, probably due to high educational pressure and limited outdoor times (5). Unawareness of appropriate myopia control approaches may aggravate ametropia and cause it to develop into high myopia, which increases the risk of retinopathy, glaucoma, cataracts, and other oculopathies (6, 7). Irreversible visual acuity damage caused by high myopia leads to loss of productivity, bringing substantial economic burden (8). Thus, the increase in myopia cases must be given great attention, and effective and safe measures must be devised and applied to slow myopia progression.

Atropine, a muscarinic acetylcholine receptor antagonist that pharmacologically retards myopia progression, has been widely studied of late. Several trials have reported the outcomes of the use of various concentrations of atropine eye drops in children and adolescents with myopia (9–11). Systematic reviews and meta-analyses have also been conducted to compare the effectiveness of the concentrations (12, 13). However, the latest clinical trial (i.e., Repka et al. (14)) about low-dose atropine turned out to be invalid in slowing myopia progression, which contradicts the results of previous studies and meta-analyses (12, 14, 15). Available studies focused on the myopia control effect of different subgroups (high dose, moderate dose, and low dose), and incomplete reported concentrations were included in the analysis. Clinical practice requires a comprehensive assessment of different concentrations of atropine used to slow myopia progression.

The examined systematic reviews and meta-analyses showed variable quality and results due to the use of different inclusion criteria and subgroup categories; hence, it is necessary to over review the quality and results of previous studies. The present umbrella review aimed to summarize the different atropine concentrations used to slow myopia progression as reported in published systematic reviews and meta-analyses, assess their tolerability and efficacy in slowing myopia progression, and determine practicable concentrations.

## 2 Methods

We performed this umbrella review to investigate the efficacy and tolerability of different concentrations of atropine in slowing the myopia progression in adolescents and children. Systematic reviews with and without meta-analyses of randomized control trials (RCTs) and observational studies were included in this review. Study protocol was registered prospectively with PROSPERO (CRD42023466785). The results were reported according to the Preferred Reporting Items for Systematic Reviews and Meta-analyses (PRISMA) protocol statement (**Supplementary Table S1**). Full details of Methods for conducting this review are presented in **Supplementary Materials**.

### 2.1 Data sources and search strategy

The present umbrella review included systematic reviews and meta-analyses of the use of atropine for myopia control in children and adolescents. Pubmed, Embase, and Cochrane Library were searched for relevant studies. Two reviewers independently searched the relevant systematic reviews and meta-analyses conducted and uploaded from the databases’ inception to August 25, 2023. The mesh terms “atropine,” “mydriatics,” “cycloplegia,” “myopia,” “systematic review,” and “meta-analysis” were used for the searches, and the articles’ references were screened to identify additional studies. The detailed search strategies used are shown in the **Supplementary Material** (**Supplementary Table S3**).

### 2.2 Inclusion and exclusion criteria

The inclusion criteria were as follows: (a) study type: systematic review or meta-analysis based on RCTs or observational studies on the topic of myopia control; (b) study participants: aged under 18 years with myopia; (c) intervention and control: the intervention group should have received atropine drops with the concentration varying from 0.01% to 1%, and the control group should have received saline or placebo, or should have been blank control (studies comparing the other myopia control approaches and atropine plus approaches were also included); and (d) report of outcomes: the main outcomes included the changes in spherical equivalent refraction (SER) or axial length (AL) expressed as weight mean difference (WMD) with 95% credible interval (95% CI).

The exclusion criteria were as follows: (a) articles reporting data regarding atropine used in animals; (b) study protocols, conference abstracts, and network meta-analyses, or articles without full texts; (c) articles reporting unavailable data; (d) studies cited incorrect data or references.

### 2.3 Review and data extraction

The identified studies in the three databases were imported into Endnote X9, and duplicate studies were eliminated. Two independent reviewers (BZ.C. and Y.N.) screened the abstracts and titles to identify and collect eligible studies. The articles’ authors and publication dates, the countries of origin of the included RCTs or observational studies, the study durations and covered years, the numbers of included studies and patients, treatment and control information, outcomes, conclusions, and data from related RCTs or observational studies were extracted. We also searched and screened the original RCTs or observational studies of the systematic reviews and meta-analyses to make sure the data and references were matched correctly.

### 2.4 Quality and evidence assessment

The methodological quality of the included systematic reviews and meta-analyses was evaluated by independent reviewers using A Measure Tool to Assess Systematic Reviews version 2 (AMSTAR 2). The critical areas were assessed by seven items (16). Each item was classified as “No,” “Partial Yes,” or “Yes” based on its conformance to the criteria. The overall level of methodological quality was categorized as “high,” “moderate,” “low,” or “critically low.” The disparities that arose throughout the evaluation were resolved through discussion by the third reviewer (ZT.Z.).

Evidence of outcomes was classified into four categories using evidence classification criteria according to previous umbrella reviews (17–20): class I (convincing evidence), class II (suggestive evidence), class III (weak evidence), and class IV (non-significant) (**Table 1**).

**Table 1:** Evidence of criteria.

Categories	Criteria
Class I: Convincing evidence	Cases number >1000 *P* <.001 *I^2^* < 50% Largest study with statistically significant effect (*P* <.05) No small-study effects 95% prediction interval excluded null value
Class II: Suggestive evidence	*P* <.001 *I^2^* < 50% Largest study with statistically significant effect (*P* <.05) No small-study effects
Class III: Weak evidence	.001 *≤ P* <.05
Class IV: Non-significant association	*P* >.05

### 2.5 Statistical analysis

A considerable number of systematic reviews and meta-analyses published within a short time frame and concentrating on the same field may contain numerous duplicate RCTs, which may introduce bias into the overall results. To evaluate the possible effect caused by including the same RCTs, we calculated the amount of overlap using the corrected covered area (CCA). The primary RCTs served as the rows in the matrix, and the included systematic reviews and meta-analyses served as the columns, as in the study by Mariam et al. (21) The total number of RCTs included in systematic reviews and meta-analyses, RCTs, and included systematic reviews and meta-analyses were denoted by “N” (repetition permitted), “r,” and “c,” respectively; then, CCA = (N − r)/[(r × c) − r]. Minor overlap was indicated by a 0% to 5% CCA value, moderate overlap by 6% to 10%, high overlap by 11% to 15%, and very high overlap by a value > 15%.

Original data from the RCTs or observational studies included in the systematic reviews and meta-analyses were extracted and reanalyzed using Revman 5.4. We calculated the annual changes in SER and AL in each study or collected the annual change data to make them comparable for the different study durations of the RCTs or observational studies. The annual mean differences were calculated by dividing the total change in mean differences by duration (years).

Using the data from the individual RCTs, we retooled various outcome indicators with incongruent systematic review and meta-analysis effect sizes. The random-effects model was used to estimate the overall effect size.

## 3 Results

**Figure 1:**
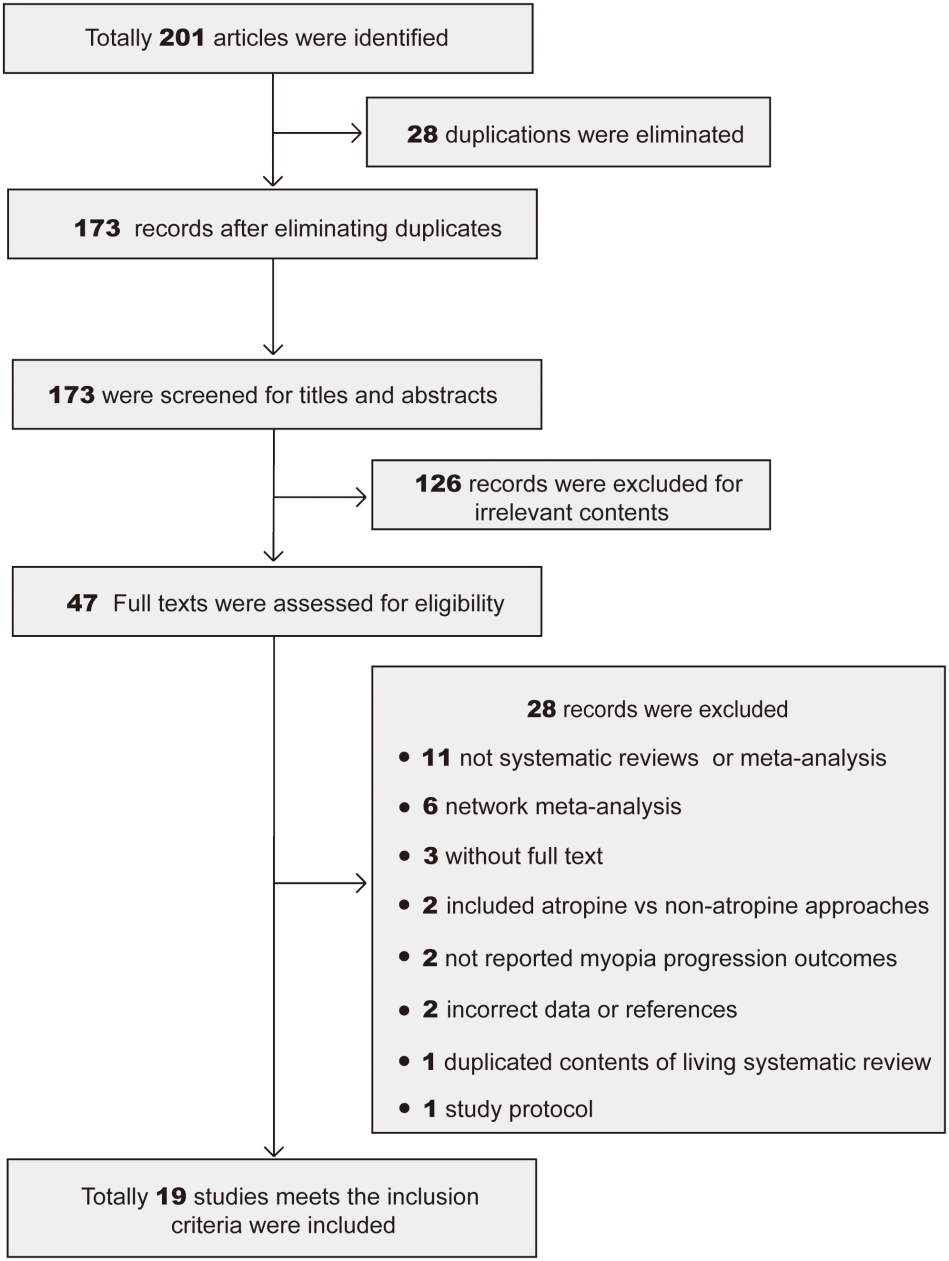
PRISMA flowchart of the study selection process. Totally 201 meta-analyses and systematic reviews were found in 3 databases. 28 duplications were eliminated, 126 articles were eliminated for irrelevant to the topic, 28 articles were eliminated due to improper or incomplete contents. Finally 19 studies were included for analyze.

Through the research strategy, 201 potentially relevant articles were obtained, 28 of which were excluded as they were duplicates. Following a title- and abstract-based screening, 126 articles were eliminated due to irrelevant content. The remaining 47 articles were then retrieved for full-text evaluation. 28 articles were eliminated. Finally, 19 articles (13, 15, 22–38) were included in the present umbrella review. The flowchart (**Figure 1**) shows the literature screening procedure.

### 3.1 Characteristics of the included studies

The characteristics of the 19 systematic reviews and meta-analyses included in the present umbrella review are summarized in **Supplementary Table S2**. There were 23 RCTs across the 19 systematic reviews and meta-analyses, and their corresponding relationships are shown in **Supplementary Table S4**. The overall CCA value was 14.14%, indicating a high level of overlap. The CCA of the studies that reported AL changes was 14.93%, indicating high overlap, while the studies that reported SER changes presented very high overlap (CCA = 18.1%). The included systematic reviews and meta-analyses were published between 2011 and 2023, and most of the original RCTs or observational studies in them focused on East Asia, India, Singapore, and the United States.

### 3.2 Quality assessment

Using AMSTAR 2 tool, we assessed the systematic reviews and meta-analyses, 1 as having high quality, 7 as having moderate quality, 7 as having low quality, and 4 as having critically low quality. Item 13 (adequate addressing of the risk of bias in studies; 13/19 of SRs/MAs, 68.42%) and Item 15 (assessment of presence and likely impact of publication bias; 4/19 of SRs/MAs, 21.05%) were the most frequently absent critical items. **Supplementary Table S5** provides the evaluation results of the AMSTAR 2 assessment for each study.

### 3.3 Myopia control effect

**Figure 2:**
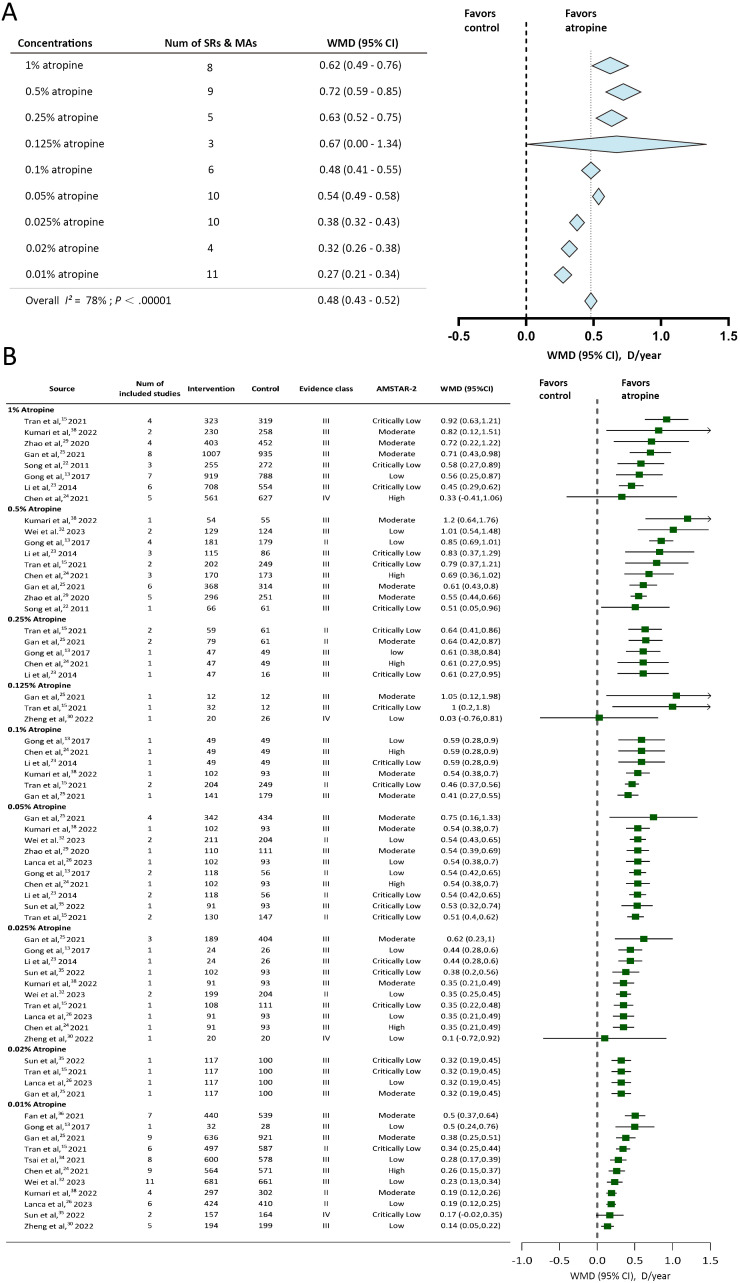
Forest plot of annual Spherical Equivalent Refraction (SER) change for myopic individuals with different concentrations of atropine vs control. **(A)** Shows the summary effect of each concentration, length of diamonds covers the 95% credible interval (CI) of summary effect sizes. **(B)** Shows effect sizes of the included systematic reviews or meta-analyses. WMD, mean difference; Num, number; D, diopter; SRs, systematic reviews; MAs, meta-analyses; AMSTAR-2, A Measurement Tool for the Assessment of Multiple Systematic Reviews version 2.

**Figure 3:**
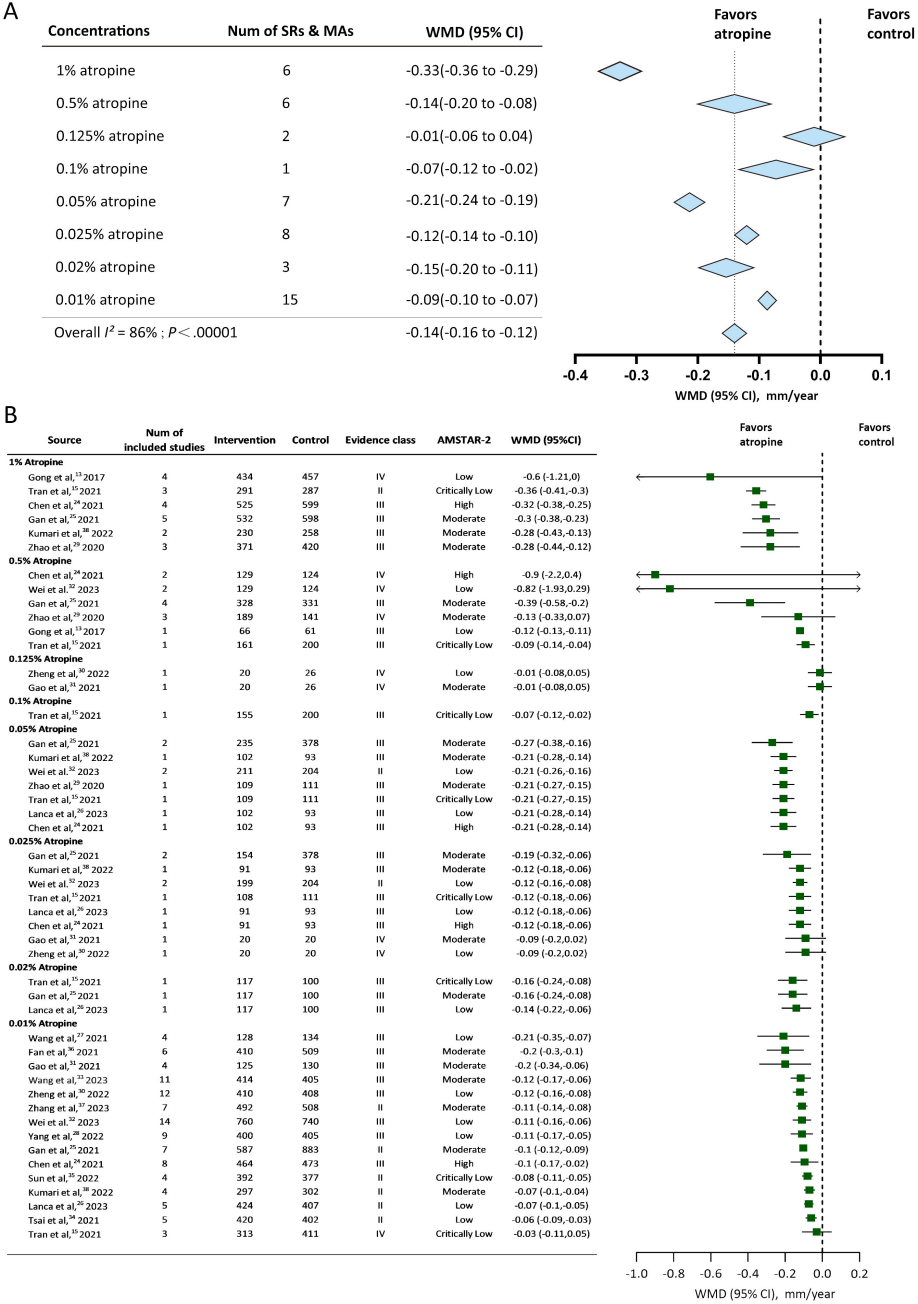
Forest plot of annual Axial Length (AL) change for myopic individuals with different concentrations of atropine vs control. **(A)** Shows the summary effect of each concentration, length of diamonds covers the 95% credible interval (CI) of summary effect sizes. **(B)** Shows effect sizes of the included systematic reviews or meta-analyses. WMD, mean difference; Num, number; mm, millimeter; SRs, systematic reviews; MAs, meta-analyses; AMSTAR-2, A Measurement Tool for the Assessment of Multiple Systematic Reviews version 2.

Available studies reported 9 atropine concentrations (0.01%, 0.02%, 0.025%, 0.05%, 0.1%, 0.125%, 0.25%, 0.5%, and 1%). SER change was reported by 73.68% (14/19) of the studies, and AL change was reported by 89.47% (17/19) of the studies. The Overall effect of annual SER change and annual AL change were shown in **Figures 2A**, **3A**. Evidence class and the number of patients in compared subgroups were shown in **Figures 2B**, **3B**.

#### 3.3.1 Spherical equivalent refraction change

The included studies reported 9 concentrations of atropine and estimated the myopia control effect using SER change. All of the concentrations showed slower SER progression compared to the control group. The overall effect of 9 concentrations of atropine is 0.48D/year (95% CI -0.43 to 0.52). 1%, 0.5%, 0.25%, 0.125% and 0.05% showed slower SER progression than the overall effect. 0.1% showed an identical annual SER change but a larger 95% credible interval compared to the overall effect. The annual SER change was less in 0.01%, 0.02% and 0.025% atropine than the overall effect. 0.5% atropine showed the maximum annual SER change among 9 concentrations (0.72D/year (95%CI 0.59 to 0.85)). Despite the 0.01% showed the minimum annual SER change among these 9 concentrations, it still showed the significant myopia control effect with an annual SER change of 0.27D/year (95% CI 0.21 to 0.34).

#### 3.3.2 Axial length change

8 concentrations of atropine reported the axial length change to estimate the myopia control effect. All of them except 0.125% showed slowing axial elongation compared to the control group. The overall effect of the annual axial length change was -0.14mm/year (95%CI -0.16 to -0.12). 1%, 0.05%, and 0.02% atropine showed slower axial elongation compared to the overall effect. 0.01%, 0.025%, 0.1% showed less annual axial length change than the overall effect. 0.5% atropine had the same WMD with the overall effect but a larger 95% credible interval. 1% atropine had the best slowing axial elongation effect -0.33mm/year (95%CI -0.36 to -0.29). 0.125% had an annual axial change of -0.01mm/year (95%CI -0.06 to 0.04), which was considered statistically insignificant (*P* = 0.67) in slowing myopia progression.

### 3.4 Tolerability

Fourteen included studies summarized and reported adverse events occurring in the original RCTs or observational studies. Photophobia, accommodation dysfunction, and blurred near vision were the most frequently reported adverse events. Allergy, headache, and systemic flushes were also mentioned in a few cases. For the studies comparing the atropine plus orthokeratology with orthokeratology, contact lens–related events, such as corneal staining, conjunctivitis, and keratitis, were reported in both the intervention and control groups. Gan et al. (25) reported that the odd ratio (OR) of photophobia was 163.57 in high-dose atropine (0.5% and 1%), 8.63 in moderate-dose atropine (0.02% to 0.25%), and 6.04 in low-dose atropine (0.01%). Gong et al. (13) reported that the incidence of photophobia was 43.8% in high-dose atropine, 17.8% in moderate-dose atropine, and 6.3% in low-dose atropine.

## 4 Discussion

The present umbrella review included systematic reviews and meta-analyses that reported data regarding the use of nine atropine concentrations (from 0.01% to 1%) for myopia control. SER and AL changes were used as outcomes to estimate the myopia control effect. The 0.01%, 0.02%, 0.025%, 0.05%, 0.1%, 0.25%, 0.5%, and 1% concentrations reported significant myopia progression–slowing effects by estimating the SER change. 1%, 0.5%, 0.25%, 0.125% and 0.05% slowed SER progression more than the average overall effect and the 0.01% 0.02% and 0.025% were less than the average level in controlling SER progression. 8 of the concentrations reported axial length change and 7 of them (except 0.125%) showed significant slowdown of axial elongation. 1%, 0.05% and 0.02% showed better efficacy than the overall effect in slowing axial elongation, while 0.01%, 0.025 and 0.1% did not. Combined with the analyzed results of annual SER change and axial length change, 0.05% and 1% showed accordant better myopia control effect than the average efficacy. 0.01% and 0.025% showed accordant less in slowing myopia progression compared to the average efficacy. The residual concentrations (0.02%, 0.1%, 0.25%, 0.5%) showed inconsistent results compared to the average efficacy in annual SER change and axial length change and 0.125% atropine had statistically insignificant effects (*P* ≥ 0.05). These may be attributed to the insufficient number of participants included and insufficient studies that had reported these concentrations.

The examined systematic reviews and meta-analyses revealed the effects of some of the atropine concentrations, albeit with some limitations present in recent studies. Several meta-analyses demonstrated the effectiveness of atropine in slowing myopia progression. Chen et al. (24) reported annual SER changes with 0.01%, 0.02% to 0.5%, and 1% atropine. Their findings indicated a statistically significant slowdown in myopia progression with 0.01% atropine (annual SER change WMD 0.26 D/year [95% CI 0.15 - 0.37]) and 0.025% to 0.5% atropine (annual SER change WMD 0.59 D/year [95% CI 0.41 - 0.78]). These results align with ours, suggesting that moderate-dose atropine is more effective than low-dose atropine in slowing myopia progression. The results reported by Gan et al. (25) and Gong et al. (13) also revealed greater myopia progression–slowing effects of high/moderate-dose atropine compared to low-dose atropine. A network meta-analysis was conducted to compare the myopia control effects of different atropine concentrations (12), and 0.05%, 0.5%, and 1% atropine exhibited favorable outcomes. However, each arm included a limited number of studies and might have had unconvincing results. The authors also acknowledged the limitation of insufficiently included studies in their analysis.

Does 0.01% atropine slow myopia progression? Repka et al. (14) reported the latest clinical trial involving the use of 0.01% atropine for myopia control, and the results indicated that 0.01% atropine did not confer benefits for myopia control, contrary to the results of numerous clinical trials and meta-analyses. The clinical trial was conducted rigorously, the refractions of the included participants were measured three times by optometrists, and no previous myopia control was permitted. Furthermore, various races (Black, East Asian, West/South Asian, Hispanic, Latino, and White) were included to prevent the influence of genetic factors. However, most of the clinical trials were conducted in East and South Asia because of the high prevalence of myopia in these regions. Repka et al. (14) ‘s trial was conducted in the United States, and only 13.9% (26/187) of the participants were Asians. The authors also reported that they carried out virtual visits via phone or video due to the coronavirus disease 2019 pandemic. Eye drops were just delivered to families, which made it challenging to ensure compliance with atropine usage. These could probably explain the divergent results regarding the efficacy of 0.01% atropine in controlling myopia. Yam et al. (9) conducted another clinical trial that reported the ineffectiveness of 0.01% atropine compared to placebo in controlling myopia. However, it is notable that the participants were children with SER between + 1.00 D and 0.00 D, which means that the research focused on the myopia prevention effect rather than the myopia control effect. The outcomes of 0.01% atropine may thus be attributed to the differences in the study populations under investigation.

Although the two aforementioned clinical trials reported the myopia control inefficacy of 0.01% atropine, most of the studies in our umbrella review reported a significant myopia progression–slowing effect. The LAMP study (39) reported an annual SER change (mean [SD]) of -0.59 (0.61) D for 0.01% atropine compared to -0.81 (0.53) D for placebo, and an annual AL change of 0.36 (0.29) mm versus 0.41 (0.22) mm. Similarly, Lee et al. (40) reported an annual SER change of -0.31 D (95% CI -0.39 to -0.22) for 0.01% atropine versus -0.53 D (95% CI -0.66 to -0.40) for placebo, and an annual AL change of 0.16 mm (95% CI 0.13 - 0.20) versus 0.25 mm (95% CI 0.20 - 0.30), which are consistent with our results.

The myopia control white paper (41) regarded an annual SER change ≥ 0.25 D/year or an annual AL change ≥ 0.1 mm/year as effective in combined therapies. According to International Myopia Institute (IMI) white papers (42), treatment effects ranging from 70% to 100% were reported with 0.5% and 1% atropine. However, the criteria for the efficacy of monotherapy are void. Due to the lack of practical guidelines for assessing the efficacy of clinical myopia control approaches, the assessment remains challenging. Guidelines for estimating myopia progression are thus needed.

To make the myopia control effect comparable and more accurate, our research standardized the estimated criterion by calculating the annual SER change and annual axial length change. Clinical practice focuses on the efficacy of specific concentrations rather than a rough comparison among concentration subgroups, so the myopia control effect of 9 previously reported concentrations of atropine was respectively reanalyzed in our study, which provides more specific evidence of each concentration in slowing myopia progression. We reanalyzed the myopia control effect based on the available systematic reviews and meta-analyses, providing a higher rank of evidence for the controversial conclusion (for example, the myopia control effect of 0.01% atropine). Furthermore, the quality and the rank of evidence were applied to show the reliability of the analysis.

There are still several limitations in our research. The present umbrella review included comprehensive systematic reviews and meta-analyses and formally assessed the quality of the included studies. More RCTs and observational studies were included and reanalyzed compared to the available meta-analyses to make the results of the review more convincing. However, our research had several limitations. First, there was a small initial number of studies included, and there were inadequate studies in some concentration groups, which may have led to inaccurate results. Second, more than half of the included studies were assessed as having low or critically low quality, increasing the risk of overestimating the effect size. Third, as some concentration groups and included studies did not report adverse events, tolerability was hard to accurately estimate in each concentration.

## 5 Conclusion

In conclusion, the present umbrella review identified and gave an overview of atropine use and myopia control outcomes. All the reported atropine concentrations showed a myopia progression alleviation effect in children and adolescents. Adverse events occur more in high-dose of atropine than in moderate and low doses of atropine. However, higher-quality multicenter studies with participants spanning a wide range of races are still needed to elucidate the dose–response relationship between atropine and myopia control, and practical guidelines are needed for assessing myopia progression control efficacy.

## Data Availability

Publicly available datasets were analyzed in this study. This data can be found here: https://github.com/krystian-scott/Re-arranged-Results.
