# Supplementary material for: Comparison of different concentrations atropine in controlling children and adolescent myopia: an umbrella review of systematic reviews and meta-analyses
Source: Front Ophthalmol (Lausanne). 2024 Oct 23;4:1447558. doi: 10.3389/fopht.2024.1447558 (PMC11537912; doi:10.3389/fopht.2024.1447558)
Supplement: Supplementary file 1 [file DataSheet1.docx]

Supplementary Material

# Supplementary Figures and Tables

**Table S1**. PRISMA 2020 Checklist

| **Section and Topic** | **Item #** | **Checklist item** | **Location where item is reported** |
| --- | --- | --- | --- |
| **TITLE** | | |  |
| Title | 1 | Identify the report as a systematic review. | P1 |
| **ABSTRACT** | | |  |
| Abstract | 2 | See the PRISMA 2020 for Abstracts checklist. | P2 |
| **INTRODUCTION** | | |  |
| Rationale | 3 | Describe the rationale for the review in the context of existing knowledge. | P4 |
| Objectives | 4 | Provide an explicit statement of the objective(s) or question(s) the review addresses. | P2 |
| **METHODS** | | |  |
| Eligibility criteria | 5 | Specify the inclusion and exclusion criteria for the review and how studies were grouped for the syntheses. | P4 |
| Information sources | 6 | Specify all databases, registers, websites, organisations, reference lists and other sources searched or consulted to identify studies. Specify the date when each source was last searched or consulted. | P3 |
| Search strategy | 7 | Present the full search strategies for all databases, registers and websites, including any filters and limits used. | Table S3 |
| Selection process | 8 | Specify the methods used to decide whether a study met the inclusion criteria of the review, including how many reviewers screened each record and each report retrieved, whether they worked independently, and if applicable, details of automation tools used in the process. | P4 |
| Data collection process | 9 | Specify the methods used to collect data from reports, including how many reviewers collected data from each report, whether they worked independently, any processes for obtaining or confirming data from study investigators, and if applicable, details of automation tools used in the process. | P3, P4 |
| Data items | 10a | List and define all outcomes for which data were sought. Specify whether all results that were compatible with each outcome domain in each study were sought (e.g. for all measures, time points, analyses), and if not, the methods used to decide which results to collect. | P4 |
|  | 10b | List and define all other variables for which data were sought (e.g. participant and intervention characteristics, funding sources). Describe any assumptions made about any missing or unclear information. | Table S2 |
| Study risk of bias assessment | 11 | Specify the methods used to assess risk of bias in the included studies, including details of the tool(s) used, how many reviewers assessed each study and whether they worked independently, and if applicable, details of automation tools used in the process. | Table S5 |
| Effect measures | 12 | Specify for each outcome the effect measure(s) (e.g. risk ratio, mean difference) used in the synthesis or presentation of results. | P6 |
| Synthesis methods | 13a | Describe the processes used to decide which studies were eligible for each synthesis (e.g. tabulating the study intervention characteristics and comparing against the planned groups for each synthesis (item #5)). | Figure 1 |
|  | 13b | Describe any methods required to prepare the data for presentation or synthesis, such as handling of missing summary statistics, or data conversions. | P5 |
|  | 13c | Describe any methods used to tabulate or visually display results of individual studies and syntheses. | Figure 2, 3 |
|  | 13d | Describe any methods used to synthesize results and provide a rationale for the choice(s). If meta-analysis was performed, describe the model(s), method(s) to identify the presence and extent of statistical heterogeneity, and software package(s) used. | P5 |
|  | 13e | Describe any methods used to explore possible causes of heterogeneity among study results (e.g. subgroup analysis, meta-regression). |  |
|  | 13f | Describe any sensitivity analyses conducted to assess robustness of the synthesized results. |  |
| Reporting bias assessment | 14 | Describe any methods used to assess risk of bias due to missing results in a synthesis (arising from reporting biases). | Table S5 |
| Certainty assessment | 15 | Describe any methods used to assess certainty (or confidence) in the body of evidence for an outcome. | Table 1 |
|  |  |  |  |
| **RESULTS** | | |  |
| Study selection | 16a | Describe the results of the search and selection process, from the number of records identified in the search to the number of studies included in the review, ideally using a flow diagram. | P6 |
|  | 16b | Cite studies that might appear to meet the inclusion criteria, but which were excluded, and explain why they were excluded. | Figure 1 |
| Study characteristics | 17 | Cite each included study and present its characteristics. | Table 1 |
| Risk of bias in studies | 18 | Present assessments of risk of bias for each included study. | Table S5 |
| Results of individual studies | 19 | For all outcomes, present, for each study: (a) summary statistics for each group (where appropriate) and (b) an effect estimate and its precision (e.g. confidence/credible interval), ideally using structured tables or plots. | Figure 2,3 |
| Results of syntheses | 20a | For each synthesis, briefly summarise the characteristics and risk of bias among contributing studies. | Figure 2,3 |
|  | 20b | Present results of all statistical syntheses conducted. If meta-analysis was done, present for each the summary estimate and its precision (e.g. confidence/credible interval) and measures of statistical heterogeneity. If comparing groups, describe the direction of the effect. | Figure 2,3 |
|  | 20c | Present results of all investigations of possible causes of heterogeneity among study results. |  |
|  | 20d | Present results of all sensitivity analyses conducted to assess the robustness of the synthesized results. |  |
| Reporting biases | 21 | Present assessments of risk of bias due to missing results (arising from reporting biases) for each synthesis assessed. |  |
| Certainty of evidence | 22 | Present assessments of certainty (or confidence) in the body of evidence for each outcome assessed. | Figure 2,3 |
| **DISCUSSION** | | |  |
| Discussion | 23a | Provide a general interpretation of the results in the context of other evidence. | P8 |
|  | 23b | Discuss any limitations of the evidence included in the review. | P10 |
|  | 23c | Discuss any limitations of the review processes used. | P10 |
|  | 23d | Discuss implications of the results for practice, policy, and future research. | P8 |
| **OTHER INFORMATION** | | |  |
| Registration and protocol | 24a | Provide registration information for the review, including register name and registration number, or state that the review was not registered. | P3 |
|  | 24b | Indicate where the review protocol can be accessed, or state that a protocol was not prepared. | P3 |
|  | 24c | Describe and explain any amendments to information provided at registration or in the protocol. | P3 |
| Support | 25 | Describe sources of financial or non-financial support for the review, and the role of the funders or sponsors in the review. | P11 |
| Competing interests | 26 | Declare any competing interests of review authors. | P11 |
| Availability of data, code and other materials | 27 | Report which of the following are publicly available and where they can be found: template data collection forms; data extracted from included studies; data used for all analyses; analytic code; any other materials used in the review. | P11 |

**Table S2. Characteristics of the Included Systematic Reviews and Meta-analyses.**

| **Studies** | **Studies types** | **No. of the included studies** | **Duration of the included studies** | **Population** | **Covered years** | **Age, years** | **Included patients’ number,**  **Intervention / control** | **Intervention** | **Outcomes** | **Adverse events**  **report** |
| --- | --- | --- | --- | --- | --- | --- | --- | --- | --- | --- |
| [Song et al,^22^](https://www.liebertpub.com/doi/10.1089/jop.2011.0017?url_ver=Z39.88-2003&rfr_id=ori%3Arid%3Acrossref.org&rfr_dat=cr_pub++0pubmed) 2011 | Meta-analysis | 6 | 6 months – 24 months | Hongkong, China  Singapore  Taiwan, | 1989 - 2008 | 5 - 15 years | 503 / 382 | 0.5%ATE vs 0.25%ATE;  1%ATE vs Blank;  1% ATE, 0.5% ATE vs Placebo；  0.5%ATE, 0.25%ATE,  0.1%ATE vs Tropicamide;  1% ATE vs Saline; | SER  AL | Photophobia;  Glare;  Blurred near vision;  Allergic blepharitis; |
| [Li et al,^23^](https://pubmed.ncbi.nlm.nih.gov/?term=Li+SM&cauthor_id=24445721)2014 | Meta-analysis | 11 | 12 months – 144 months | Hongkong, China  Singapore  Taiwan  United States | 1984 - 2011 | 5 – 15 years | 1061/771 | 1% ATE vs Saline;  0.1ATE, 0.25%ATE, 0.5%ATE vs 0.5% Tropicamide;  1% ATE vs placebo;  0.05%ATE, 0.025%ATE, 1% ATE vs Blank;  0.5%ATE+Multi-focal vs Multi-focal; | SER | Not reported |
| [Chen](https://pubmed.ncbi.nlm.nih.gov/?term=Chen+C&cauthor_id=34925913) et al,^24^ 2021 | Meta-analysis | 17 | 6 months – 60 months | Singapore  Spain  Japan  India  Taiwan  Hongkong, China  China Mainland | 1989 - 2021 | 4 – 14 years | 1585/1373 | 1%ATE, 0.5%ATE, 0.05%ATE, 0.025% ATE, 0.01%ATE vs placebo;  0.5%ATE, 0.25%ATE, 0.1%ATE vs Tropicamide;  0.01%ATE+OK vs OK;  0.5%ATE+Multi-focal vs Multi-focal;  0.01%ATE+spectacles vs spectacles; | SER  AL  BCVA  ACD  LT  IOP  T-BUT | Accommodation dysfunction;  Photopic pupil size |
| [Gan](https://pubmed.ncbi.nlm.nih.gov/?term=Gan+J&cauthor_id=35096861) et al,^25^ 2022 | Meta-analysis | 27 | 12 months – 144 months | Taiwan,  Singapore  China Mainland  India  USA  Europe  Korea  Hongkong, China | 1984 - 2021 | 4 – 15 years | 2975/3499 | 0.5%ATE+Multi-focal vs Multi-focal;  1%ATE, 0.5%ATE, 0.125%ATE, 0.05%ATE, 0.025%ATE, 0.01%ATE vs placebo/blank;  1%ATE, 0.05%ATE, 0.025%ATE, 0.01%ATE vs self-contrast | SER  AL | Photophobia;  Blurred near vision;  Allergy; |
| [Gong](https://pubmed.ncbi.nlm.nih.gov/?term=Gong+Q&cauthor_id=28494063) et al,^13^ 2017 | Meta-analysis | 19 | 6 months – 144 months | Taiwan  Hongkong, China  China Mainland  USA  Singapore | 1984 - 2015 | 6 – 17 years | 1100/967 | 1%ATE, 0.5%ATE, 0.25ATE, 0.1%ATE, 0.125%ATE, 0.05%ATE, 0.025%ATE,  0.01%ATE vs Placebo/Blank;  0.5%ATE+Multi-focal vs Multi-focal; | SER  AL | Photophobia;  Poor near visual acuity;  Allergy |
| [Lanca](https://pubmed.ncbi.nlm.nih.gov/?term=Lanca+C&cauthor_id=37033047) et al,^26^ 2023 | Systematic review | 5 | 12 months -24 months | China, Mainland  Japan  India | 2018 - 2022 | 4 – 14 years | 734/696 | 0.01%ATE vs Placebo;  0.01%ATE, 0.02%ATE vs spectacles; | SER  AL | Not reported |
| [Tran](https://pubmed.ncbi.nlm.nih.gov/?term=Tran+HDM&cauthor_id=34456234) et al,^15^ 2021 | Meta-analysis | 13 | 12 months – 24 months | Hongkong, China  Taiwan  Singapore  Italia  USA  China Mainland | 1989 - 2019 | 4 – 16 years | 1672/1835 | 1%ATE, 0.5%ATE, 0.25ATE, 0.1%ATE, 0.125%ATE, 0.05%ATE, 0.025%ATE,  0.01%ATE vs Placebo/Blank; | SER  AL  AA  PD | Photophobia  Near work problem |
| [Wang](https://pubmed.ncbi.nlm.nih.gov/?term=Wang+S&cauthor_id=32781450) et al,^27^ 2020 | Meta-analysis | 4 | 1 month – 12 months | Japan  Hongkong, China  China Mainland | 2017 - 2019 | 6 – 16 years | 128/134 | 0.01%ATE + OK vs OK | AL  UCVA  CECD  IOP | Coneal staining;  Conjunctivitis;  Photophobia |
| [Yang](https://pubmed.ncbi.nlm.nih.gov/?term=Yang+N&cauthor_id=33678055) et al,^28^ 2022 | Meta-analysis | 8 | 1 month – 24 months | China Mainland  Japan | 2018 - 2020 | 5.6 – 17 years | 229/232 | 0.01%ATE + OK vs OK | AL | OK lens related adverse events |
| [Zhao](https://pubmed.ncbi.nlm.nih.gov/?term=Zhao+C&cauthor_id=33287746) et al,^29^ 2020 | Meta-analysis | 10 | 12 months – 36 months | Singapore  Taiwan  Europeans  China Mainland | 1989 - 2018 | 4 – 18 years | 809/814 | 1%ATE, 0.5%ATE, 0.25%ATE, 0.1%ATE, 0.05%ATE, 0.025%ATE, 0.01%ATE vs placebo;  0.5% ATE + Multi-focal vs Multi-focal | SER  AL | Allergy;  Glare;  Blurred near vision;  Logistical difficulties;  Photophobia;  Headaches;  Systemic flushes |
| [Zheng](https://pubmed.ncbi.nlm.nih.gov/?term=Zheng+NN&cauthor_id=35919929) et al,^30^ 2022 | Meta-analysis | 15 | 2 months – 24 months | China Mainland  Taiwan  Japan  Hongkong, China | 2017 - 2021 | 5 – 16 years | 479/490 | 0.01%ATE + OK vs OK | SER; AL  PD; AA  IOP  T-BUT  CECD | Photophobia;  Corneal staining;  Conjunctivitis; |
| [Gao et al,^31^](https://journals.lww.com/claojournal/pages/articleviewer.aspx?year=2021&issue=02000&article=00007&type=Fulltext) 2021 | Meta-analysis | 5 | 1 month – 24 months | Japan  China  Hongkong, China  Taiwan | 2017 - 2019 | 5.6 – 17 years | 177/193 | 0.01%ATE + OK vs OK;  0.125%ATE + OK vs OK;  0.025%ATE + OK vs OK | AL | Photophobia;  decreased visual acuity; allergic reactions; |
| [Wei](https://pubmed.ncbi.nlm.nih.gov/?term=Wei+XL&cauthor_id=37602338) et al,^32^ 2023 | Meta-analysis | 15 | 1 month – 24 months | Taiwan  China  Japan  Hongkong, China  India | 2001 - 2022 | 4 – 14 years | 1404/780 | 0.5%ATE + Multi-focal vs Multi-focal;  0.5%ATE, 0.05%ATE, 0.025%ATE, 0.02%ATE, 0.01%ATE vs Placebo;  0.01%ATE + OK vs OK  0.01%ATE + Spectacles vs Spectacles | SER  AL  PD  AA | Conjunctivitis;  Keratitis;  Allergy;  Light sensitivity;  Photophobia;  Near-distance vision impairment |
| [Wang et al,^33^](https://journals.plos.org/plosone/article?id=10.1371/journal.pone.0282286) 2023 | Meta-analysis | 10 | 6 months – 24 months | China  Japan | 2017 - 2022 | 6 - 18 years | 396/392 | 0.01%ATE + OK vs OK | AL; SER  PD; AA  T-BUT  LLT  CECD  UCVA  BCVA | Photophobia;  Blurred vision;  Allergic reactions; |
| [Tsai](https://www.mdpi.com/2077-0383/10/17/3766) et al,^34^ 2021 | Meta-analysis | 8 | 12 months – 24 months | China  Japan  USA  India  Italy  Hongkong, China | 2015 - 2021 | 4 - 16 years | 597/577 | 0.01%ATE vs Placebo | SER  AL  AA  PD | Poor near visual acuity;  Allergic conjunctivitis |
| [Sun](https://pubmed.ncbi.nlm.nih.gov/?term=Sun+W&cauthor_id=36123161) et al,^35^ 2022 | Meta-analysis | 5 | 12 months – 24 months | China  Hongkong, China  Japan | 2019 - 2021 | 4 – 14 years | 412/397 | 0.05%ATE, 0.025%ATE, 0.01%ATE vs Placebo;  0.02%ATE, 0.01% ATE vs Spectacles | SER  AL | Not reported |
| [Kumari et al,^38^](https://pubmed.ncbi.nlm.nih.gov/?term=Kumari+S&cauthor_id=36993096)2022 | Meta-analysis | 7 | 12 months – 24 months | Singapore  Hongkong, China  China  Japan  India | 2006 - 2021 | 4 - 14 years | 765/624 | 1%ATE, 0.5%ATE, 0.05%ATE, 0.025%ATE, 0.01%ATE vs Placebo | SER  AL | Photophobia;  Reduced accommodation amplitude;  Blurred vision |
| [Zhang et al,^37^](https://www.sciencedirect.com/science/article/abs/pii/S0181551223000281) 2023 | Meta-analysis | 7 | 4 months – 12 months | Hongkong, China  China | 2018 - 2020 | 4 - 17 years | 492/508 | 0.01% ATE vs Placebo | AL | Photophobia |
| [Fan et al,^36^](http://ies.ijo.cn/gjykcn/article/abstract/202105022?st=search) 2021 | Meta-analysis | 7 | 4 months – 24 months | China  Singapore  Hongkong, China | 2012 - 2020 | 6 - 15 years | 440/539 | 0.01% ATE vs Placebo  0.01% ATE+SVL vs SVL | SER  AL | Not reported |

Abbreviation：ATE atropine, OK orthokeratology, Multi-focol multi-focol contact lens, SVL single vision lenses, SER spherical equivalent refraction, AL axial length, BCVA best corrected visual acuity, ACD anterior chamber depth, LT lens thickness, IOP intraocular pressure, T-BUT tear film break-up time,

AA accommodation amplitude, PD pupillary diameter, UCVA uncorrected visual acuity, CECD corneal endothelial cell density, LLT lipid layer thickness

**Table S3**. Searching strategies in three databases.

| Database | Strategy for searching included studies |
| --- | --- |
| Pubmed | (("Atropine"[MeSH Terms] OR (("pharmacology"[MeSH Terms] OR "pharmacology"[All Fields] OR "pharmacologies"[All Fields] OR "pharmacology"[MeSH Subheading]) AND ("therapeutics"[MeSH Terms] OR "therapeutics"[All Fields] OR "therapies"[All Fields] OR "therapy"[MeSH Subheading] OR "therapy"[All Fields] OR "therapy s"[All Fields] OR "therapys"[All Fields])) OR (("biopharmaceutics"[MeSH Terms] OR "biopharmaceutics"[All Fields] OR "pharmaceutic"[All Fields] OR "pharmaceutics"[All Fields] OR "pharmaceutical preparations"[MeSH Terms] OR ("pharmaceutical"[All Fields] AND "preparations"[All Fields]) OR "pharmaceutical preparations"[All Fields] OR "pharmaceutical"[All Fields] OR "pharmaceuticals"[All Fields] OR "pharmaceutical s"[All Fields] OR "pharmaceutically"[All Fields]) AND ("therapeutics"[MeSH Terms] OR "therapeutics"[All Fields] OR "therapies"[All Fields] OR "therapy"[MeSH Subheading] OR "therapy"[All Fields] OR "therapy s"[All Fields] OR "therapys"[All Fields])) OR ("ophthalmic solutions"[Pharmacological Action] OR "ophthalmic solutions"[MeSH Terms] OR ("ophthalmic"[All Fields] AND "solutions"[All Fields]) OR "ophthalmic solutions"[All Fields] OR ("eye"[All Fields] AND "drops"[All Fields]) OR "eye drops"[All Fields])) AND ("Myopia"[MeSH Terms] OR (("short"[All Fields] OR "shorts"[All Fields]) AND "eyesight"[All Fields]) OR ("Myopia"[MeSH Terms] OR "Myopia"[All Fields] OR ("near"[All Fields] AND "vision"[All Fields]) OR "near vision"[All Fields]))) AND (meta-analysis[Filter] OR systematicreview[Filter]) |
| Embase | (('myopia' OR 'near vision' OR 'short eyesight') AND ('atropine' OR 'ophthalmic solution' OR 'eyedrops' OR 'mydriatic' OR 'accommodation paralysis' OR 'pharmaceutical therapy') AND ('meta analysis'/de OR 'systematic review'/de)) |
| Cochrane Library | (atropine) OR (ophthalmic solution) OR (eyedrops) OR (pharmaceutical) AND (myopia) (Word variations have been searched) |

**Table S4**. Overlapping of the included systematic reviews and meta-analysis

|  | Systematic reviews and meta-analyses | | | | | | | | | | | | | | | | | | |
| --- | --- | --- | --- | --- | --- | --- | --- | --- | --- | --- | --- | --- | --- | --- | --- | --- | --- | --- | --- |
| **Original research** | [Song et al, 2011](https://www.liebertpub.com/doi/10.1089/jop.2011.0017?url_ver=Z39.88-2003&rfr_id=ori%3Arid%3Acrossref.org&rfr_dat=cr_pub++0pubmed) | [Li et al,](https://pubmed.ncbi.nlm.nih.gov/?term=Li+SM&cauthor_id=24445721)2014 | [Chen](https://pubmed.ncbi.nlm.nih.gov/?term=Chen+C&cauthor_id=34925913) et al, 2021 | [Gan](https://pubmed.ncbi.nlm.nih.gov/?term=Gan+J&cauthor_id=35096861) et al, 2022 | [Gong](https://pubmed.ncbi.nlm.nih.gov/?term=Gong+Q&cauthor_id=28494063) et al, 2017 | [Lanca](https://pubmed.ncbi.nlm.nih.gov/?term=Lanca+C&cauthor_id=37033047) et al, 2023 | [Tran](https://pubmed.ncbi.nlm.nih.gov/?term=Tran+HDM&cauthor_id=34456234) et al, 2021 | [Yang](https://pubmed.ncbi.nlm.nih.gov/?term=Yang+N&cauthor_id=33678055) et al, 2022 | [Zhao](https://pubmed.ncbi.nlm.nih.gov/?term=Zhao+C&cauthor_id=33287746) et al, 2020 | [Zheng](https://pubmed.ncbi.nlm.nih.gov/?term=Zheng+NN&cauthor_id=35919929) et al, 2022 | [Gao et al, 2021](https://journals.lww.com/claojournal/pages/articleviewer.aspx?year=2021&issue=02000&article=00007&type=Fulltext) | [Wei](https://pubmed.ncbi.nlm.nih.gov/?term=Wei+XL&cauthor_id=37602338) et al, 2023 | [Wang](https://pubmed.ncbi.nlm.nih.gov/?term=Wang+S&cauthor_id=32781450) et al, 2020 | [Wang et al, 2023](https://journals.plos.org/plosone/article?id=10.1371/journal.pone.0282286) | [Tsai](https://www.mdpi.com/2077-0383/10/17/3766) et al, 2021 | [Sun](https://pubmed.ncbi.nlm.nih.gov/?term=Sun+W&cauthor_id=36123161) et al, 2022 | [Kumari et al,](https://pubmed.ncbi.nlm.nih.gov/?term=Kumari+S&cauthor_id=36993096)2022 | [Fan et al, 2021](http://ies.ijo.cn/gjykcn/article/abstract/202105022?st=search) | [Zhang et al, 2023](https://www.sciencedirect.com/science/article/abs/pii/S0181551223000281) |
| [Yen et al. 1989](https://pubmed.ncbi.nlm.nih.gov/2742290/)[1] | 1 | 1 | 1 | 1 | 1 | 0 | 1 | 0 | 1 | 0 | 0 | 0 | 0 | 0 | 0 | 0 | 0 | 0 | 0 |
| [Shih et al. 1999](https://pubmed.ncbi.nlm.nih.gov/10048351/)[2] | 1 | 1 | 1 | 1 | 1 | 0 | 1 | 0 | 1 | 0 | 0 | 0 | 0 | 0 | 0 | 0 | 0 | 0 | 0 |
| [Chua et al. 2006](https://pubmed.ncbi.nlm.nih.gov/16996612/)[3] | 1 | 1 | 1 | 1 | 1 | 0 | 1 | 0 | 1 | 0 | 0 | 0 | 0 | 0 | 0 | 0 | 1 | 0 | 0 |
| [Chia et al. 2012](https://pubmed.ncbi.nlm.nih.gov/21963266/)[4] | 0 | 0 | 0 | 1 | 1 | 0 | 1 | 0 | 0 | 0 | 0 | 0 | 0 | 0 | 0 | 0 | 0 | 1 | 0 |
| [Yi et al. 2015](https://pubmed.ncbi.nlm.nih.gov/26228967/)[5] | 0 | 0 | 1 | 1 | 1 | 0 | 1 | 0 | 1 | 0 | 0 | 0 | 0 | 0 | 0 | 0 | 1 | 0 | 0 |
| [Wang et al. 2017](https://pubmed.ncbi.nlm.nih.gov/28682887/)[6] | 0 | 0 | 1 | 1 | 0 | 0 | 0 | 0 | 1 | 0 | 0 | 1 | 0 | 0 | 0 | 0 | 1 | 0 | 0 |
| [Yam 2019](https://pubmed.ncbi.nlm.nih.gov/30514630/)[7] | 0 | 0 | 1 | 1 | 0 | 1 | 1 | 0 | 1 | 0 | 0 | 1 | 0 | 0 | 1 | 1 | 1 | 1 | 1 |
| [Yam et al. 2020](https://pubmed.ncbi.nlm.nih.gov/32019700/)[8] | 0 | 0 | 0 | 0 | 0 | 1 | 0 | 0 | 0 | 0 | 0 | 0 | 0 | 0 | 0 | 0 | 0 | 0 | 0 |
| [Yam et al. 2022](https://pubmed.ncbi.nlm.nih.gov/34627809/)[9] | 0 | 0 | 0 | 0 | 0 | 1 | 0 | 0 | 0 | 0 | 0 | 0 | 0 | 0 | 0 | 0 | 0 | 0 | 0 |
| [Wei et al. 2020](https://pubmed.ncbi.nlm.nih.gov/33001210/)[10] | 0 | 0 | 1 | 1 | 0 | 1 | 0 | 0 | 0 | 0 | 0 | 1 | 0 | 0 | 1 | 1 | 0 | 0 | 1 |
| [Zhu et al. 2020](https://pubmed.ncbi.nlm.nih.gov/32038101/)[11] | 0 | 0 | 0 | 1 | 0 | 0 | 0 | 0 | 0 | 0 | 0 | 0 | 0 | 0 | 0 | 0 | 0 | 0 | 0 |
| [Saxena et al. 2021](https://pubmed.ncbi.nlm.nih.gov/33545170/)[12] | 0 | 0 | 1 | 1 | 0 | 1 | 0 | 0 | 0 | 0 | 0 | 1 | 0 | 0 | 1 | 0 | 1 | 0 | 0 |
| [Hieda et al. 2021](https://pubmed.ncbi.nlm.nih.gov/33586090/)[13] | 0 | 0 | 1 | 1 | 0 | 1 | 0 | 0 | 0 | 0 | 0 | 1 | 0 | 0 | 1 | 1 | 1 | 0 | 0 |
| [Bedrossian et al. 1985](https://pubmed.ncbi.nlm.nih.gov/4011150/)[14] | 0 | 0 | 0 | 1 | 0 | 0 | 0 | 0 | 0 | 0 | 0 | 0 | 0 | 0 | 0 | 0 | 0 | 0 | 0 |
| [Chou et al. 1997](https://pubmed.ncbi.nlm.nih.gov/9029440/)[15] | 0 | 1 | 0 | 1 | 1 | 0 | 0 | 0 | 0 | 0 | 0 | 0 | 0 | 0 | 0 | 0 | 0 | 0 | 0 |
| [Kennedy et al. 2000](https://pubmed.ncbi.nlm.nih.gov/11486796/)[16] | 0 | 1 | 0 | 1 | 1 | 0 | 0 | 0 | 0 | 0 | 0 | 0 | 0 | 0 | 0 | 0 | 0 | 0 | 0 |
| [Lee et al. 2006](https://pubmed.ncbi.nlm.nih.gov/16503774/)[17] | 0 | 1 | 0 | 1 | 1 | 0 | 1 | 0 | 0 | 0 | 0 | 0 | 0 | 0 | 0 | 0 | 0 | 0 | 0 |
| [Fan et al. 2007](https://pubmed.ncbi.nlm.nih.gov/17295137/)[18] | 1 | 1 | 0 | 1 | 1 | 0 | 1 | 0 | 0 | 0 | 0 | 0 | 0 | 0 | 0 | 0 | 0 | 0 | 0 |
| [Fang et al. 2010](https://pubmed.ncbi.nlm.nih.gov/20698798/)[19] | 0 | 1 | 0 | 1 | 1 | 0 | 0 | 0 | 0 | 0 | 0 | 0 | 0 | 0 | 0 | 0 | 0 | 0 | 0 |
| [Wu et al. 2011](https://pubmed.ncbi.nlm.nih.gov/21815829/)[20] | 0 | 1 | 0 | 1 | 1 | 0 | 0 | 0 | 0 | 0 | 0 | 0 | 0 | 0 | 0 | 0 | 0 | 0 | 0 |
| [Lin et al. 2013](https://pubmed.ncbi.nlm.nih.gov/24212187/)[21] | 0 | 0 | 0 | 1 | 1 | 0 | 0 | 0 | 0 | 0 | 0 | 0 | 0 | 0 | 0 | 0 | 0 | 0 | 0 |
| [Clark et al. 2015](https://pubmed.ncbi.nlm.nih.gov/26218150/)[22] | 0 | 0 | 0 | 1 | 1 | 0 | 1 | 0 | 0 | 0 | 0 | 0 | 0 | 0 | 1 | 0 | 0 | 0 | 0 |
| [Lee et al. 2016](https://pubmed.ncbi.nlm.nih.gov/27435576/)[23] | 0 | 0 | 0 | 1 | 0 | 0 | 1 | 0 | 0 | 0 | 0 | 0 | 0 | 0 | 0 | 0 | 0 | 0 | 0 |
| [Polling et al. 2020](https://pubmed.ncbi.nlm.nih.gov/32958872/)[24] | 0 | 0 | 0 | 1 | 0 | 0 | 0 | 0 | 1 | 0 | 0 | 0 | 0 | 0 | 0 | 0 | 0 | 0 | 0 |
| [Moon et al. 2018](https://pubmed.ncbi.nlm.nih.gov/30364238/)[25] | 0 | 0 | 0 | 1 | 0 | 0 | 0 | 0 | 0 | 0 | 0 | 0 | 0 | 0 | 0 | 0 | 0 | 0 | 0 |
| [Larkin et al. 2019](https://pubmed.ncbi.nlm.nih.gov/31602553/)[26] | 0 | 0 | 0 | 1 | 0 | 0 | 1 | 0 | 0 | 0 | 0 | 0 | 0 | 0 | 1 | 0 | 0 | 0 | 0 |
| [Sacchi et al. 2019](https://pubmed.ncbi.nlm.nih.gov/31197953/)[27] | 0 | 0 | 0 | 1 | 0 | 0 | 1 | 0 | 0 | 0 | 0 | 0 | 0 | 0 | 1 | 0 | 0 | 0 | 0 |
| [Fu et al. 2020](https://pubmed.ncbi.nlm.nih.gov/32086237/)[28] | 0 | 0 | 0 | 1 | 0 | 1 | 1 | 0 | 0 | 0 | 0 | 0 | 0 | 0 | 1 | 1 | 0 | 0 | 0 |
| [Liang et al. 2008](https://pubmed.ncbi.nlm.nih.gov/19028329/)[29] | 1 | 0 | 0 | 0 | 0 | 0 | 0 | 0 | 0 | 0 | 0 | 0 | 0 | 0 | 0 | 0 | 0 | 0 | 0 |
| [Hsiao et al. 2005](https://pubmed.ncbi.nlm.nih.gov/16206249/)[30] | 1 | 0 | 0 | 0 | 1 | 0 | 0 | 0 | 1 | 0 | 0 | 0 | 0 | 0 | 0 | 0 | 0 | 0 | 0 |
| [Shih et al. 2001](https://pubmed.ncbi.nlm.nih.gov/11401629/)[31] | 0 | 1 | 1 | 1 | 1 | 0 | 0 | 0 | 1 | 0 | 0 | 1 | 0 | 0 | 0 | 0 | 0 | 0 | 0 |
| [Brodstein et al. 1984](https://pubmed.ncbi.nlm.nih.gov/6514306/)[32] | 0 | 1 | 0 | 0 | 0 | 0 | 0 | 0 | 0 | 0 | 0 | 0 | 0 | 0 | 0 | 0 | 0 | 0 | 0 |
| [Diaz-Llopis et al. 2018](https://pubmed.ncbi.nlm.nih.gov/29398233/)[33] | 0 | 0 | 1 | 0 | 0 | 0 | 0 | 0 | 0 | 0 | 0 | 0 | 0 | 0 | 0 | 0 | 0 | 0 | 0 |
| [Kumaran et al. 2015](https://pubmed.ncbi.nlm.nih.gov/26313301/)[34] | 0 | 0 | 1 | 0 | 1 | 0 | 0 | 0 | 1 | 0 | 0 | 0 | 0 | 0 | 0 | 0 | 0 | 0 | 0 |
| [Tan et al. 2020](https://pubmed.ncbi.nlm.nih.gov/32776533/)[35] | 0 | 0 | 1 | 0 | 0 | 0 | 0 | 1 | 0 | 1 | 0 | 1 | 0 | 1 | 0 | 0 | 0 | 0 | 0 |
| [Tang et al. 2020](https://search.bvsalud.org/gim/resource/en/wpr-876809)[36] | 0 | 0 | 1 | 0 | 0 | 0 | 0 | 0 | 0 | 0 | 0 | 0 | 0 | 1 | 0 | 0 | 0 | 0 | 0 |
| [Tong et al. 2009](https://pubmed.ncbi.nlm.nih.gov/19167081/)[37] | 0 | 0 | 1 | 0 | 0 | 0 | 0 | 0 | 0 | 0 | 0 | 0 | 0 | 0 | 0 | 0 | 0 | 0 | 0 |
| [Zhao et al. 2021](https://pubmed.ncbi.nlm.nih.gov/33205372/)[38] | 0 | 0 | 1 | 0 | 0 | 0 | 0 | 0 | 0 | 1 | 0 | 1 | 0 | 1 | 0 | 1 | 0 | 0 | 0 |
| [Lin et al. 2014](https://pubmed.ncbi.nlm.nih.gov/24685184/)[39] | 0 | 0 | 0 | 0 | 1 | 0 | 0 | 0 | 0 | 0 | 0 | 0 | 0 | 0 | 0 | 0 | 0 | 0 | 0 |
| [Chen et al. 2019](https://pubmed.ncbi.nlm.nih.gov/30482510/)[40] | 0 | 0 | 0 | 0 | 0 | 0 | 0 | 1 | 0 | 1 | 1 | 0 | 0 | 1 | 0 | 0 | 0 | 0 | 0 |
| [Wan et al. 2018](https://pubmed.ncbi.nlm.nih.gov/30205439/)[41] | 0 | 0 | 0 | 0 | 0 | 0 | 0 | 1 | 0 | 0 | 1 | 0 | 0 | 0 | 0 | 0 | 0 | 0 | 0 |
| [Tan et al. 2019](https://pubmed.ncbi.nlm.nih.gov/30632410/)[42] | 0 | 0 | 0 | 0 | 0 | 0 | 0 | 1 | 0 | 0 | 1 | 1 | 1 | 1 | 0 | 0 | 0 | 0 | 0 |
| [Kinoshita et al. 2018](https://pubmed.ncbi.nlm.nih.gov/29974278/)[43] | 0 | 0 | 0 | 0 | 0 | 0 | 0 | 1 | 0 | 1 | 1 | 1 | 1 | 1 | 0 | 0 | 0 | 0 | 0 |
| [Vincent et al. 2020](https://pubmed.ncbi.nlm.nih.gov/32888318/)[44] | 0 | 0 | 1 | 0 | 0 | 0 | 0 | 1 | 0 | 1 | 0 | 0 | 0 | 1 | 0 | 0 | 0 | 0 | 0 |
| [Kinoshita et al. 2020](https://pubmed.ncbi.nlm.nih.gov/32728111/)[45] | 0 | 0 | 0 | 0 | 0 | 0 | 0 | 1 | 0 | 1 | 0 | 1 | 0 | 1 | 0 | 0 | 0 | 0 | 0 |
| [Zhao et al. 2021](https://pubmed.ncbi.nlm.nih.gov/32620344/)[46] | 0 | 0 | 0 | 0 | 0 | 0 | 0 | 1 | 0 | 1 | 0 | 0 | 0 | 0 | 0 | 0 | 0 | 0 | 0 |
| [Luo et al. 2021](https://pesquisa.bvsalud.org/portal/resource/pt/wpr-837714)[47] | 0 | 0 | 0 | 0 | 0 | 0 | 0 | 0 | 0 | 1 | 0 | 0 | 0 | 1 | 0 | 0 | 0 | 0 | 0 |
| [Zhou et al. 2021](https://pubmed.ncbi.nlm.nih.gov/34228946/)[48] | 0 | 0 | 0 | 0 | 0 | 0 | 0 | 0 | 0 | 1 | 0 | 0 | 0 | 0 | 0 | 0 | 0 | 0 | 0 |
| [Chen et al. 2020](https://pubmed.ncbi.nlm.nih.gov/33707188/)[49] | 0 | 0 | 0 | 0 | 0 | 0 | 0 | 0 | 0 | 1 | 0 | 0 | 0 | 1 | 0 | 0 | 0 | 0 | 0 |
| [Shi et al. 2017](https://kns.cnki.net/kcms2/article/abstract?v=kMpVSI0yL5e4WybC_Vkk2fuxkVrH5GbNYw_YTm1kh7OPigGpkPAH-gHEf-rjEIMXPQPBdZs9TLhRZrzjmdBzM-L9mFD2ZGjBHkSE8_x4dFc-NOsldYf2li52FIC1Dyasy7D-V1XxCz0zK3wcd6j3kA==&uniplatform=NZKPT)[50] | 0 | 0 | 0 | 0 | 0 | 0 | 0 | 0 | 0 | 1 | 1 | 0 | 1 | 0 | 0 | 0 | 0 | 0 | 0 |
| [Hao et al. 2021](https://pubmed.ncbi.nlm.nih.gov/33954859/)[51] | 0 | 0 | 0 | 0 | 0 | 0 | 0 | 0 | 0 | 1 | 0 | 0 | 0 | 0 | 0 | 0 | 0 | 0 | 0 |
| [Zhao et al. 2021](https://kns.cnki.net/kcms2/article/abstract?v=kMpVSI0yL5dQ4276iV5pAyfWU3n2DY2HKMzFSZMTqpB6GY8slqNSonSaLil2zTGIgiuCuS4EkUTEm1HfM22w1Od2jLTcswWIIrnVdoclpgs427ALRXZR22QTM90sIB8YtB8XIPTPdLQDikTeVgtcPw==&uniplatform=NZKPT)[52] | 0 | 0 | 0 | 0 | 0 | 0 | 0 | 0 | 0 | 1 | 0 | 0 | 0 | 0 | 0 | 0 | 0 | 0 | 0 |
| [Zhang et al. 2021](https://kns.cnki.net/kcms2/article/abstract?v=kMpVSI0yL5ddlNX5yFtpkyWLUHClEJiHZkSNwalTqzQbAzbueT_YFhcKk1dbmNRRsmIVIUPtb0V3BbuB6pxmzOQmEEbFkRAQtu-W9QyGIDMUPMm2GMJY9--hKgY5YPIzLlblg8lPBDtEXsPmMriENw==&uniplatform=NZKPT)[53] | 0 | 0 | 0 | 0 | 0 | 0 | 0 | 0 | 0 | 1 | 0 | 0 | 0 | 0 | 0 | 0 | 0 | 0 | 0 |
| [Cui et al. 2021](https://pubmed.ncbi.nlm.nih.gov/34782708/)[54] | 0 | 0 | 0 | 0 | 0 | 0 | 0 | 0 | 0 | 0 | 0 | 1 | 0 | 0 | 0 | 0 | 0 | 0 | 0 |
| [Wang et al. 2022](https://pubmed.ncbi.nlm.nih.gov/35184254/)[55] | 0 | 0 | 0 | 0 | 0 | 0 | 0 | 0 | 0 | 0 | 0 | 1 | 0 | 0 | 0 | 0 | 0 | 0 | 0 |
| [Shi 2018](https://kns.cnki.net/kcms2/article/abstract?v=kMpVSI0yL5fzAxa2ytZjc7fh4NUgK0SkJQimfjeBWdJZ9Fr6_i-qQdZ3tCaW45k_gb_BdBH58-x135zVRF_fXLiUQ2KtuYZCZEBb55OyghU-dqMziLiAFBGrJn805YcffTThmIv36t0=&uniplatform=NZKPT)[56] | 0 | 0 | 0 | 0 | 0 | 0 | 0 | 0 | 0 | 0 | 0 | 0 | 1 | 0 | 0 | 0 | 0 | 0 | 0 |
| [Ji et al. 2022](https://pubmed.ncbi.nlm.nih.gov/34924543/)[57] | 0 | 0 | 0 | 0 | 0 | 0 | 0 | 0 | 0 | 0 | 0 | 0 | 0 | 1 | 0 | 0 | 0 | 0 | 0 |
| [Jiang 2018](http://ies.ijo.cn/gjykcn/article/abstract/201807046)[58] | 0 | 0 | 0 | 0 | 0 | 0 | 0 | 0 | 0 | 0 | 0 | 0 | 0 | 1 | 0 | 0 | 0 | 0 | 0 |
| [Ren et al. 2017](http://ies.ijo.cn/gjykcn/article/abstract/201704057)[59] | 0 | 0 | 0 | 0 | 0 | 0 | 0 | 0 | 0 | 0 | 0 | 0 | 0 | 1 | 0 | 0 | 0 | 1 | 0 |
| [Niu et al. 2019](http://ies.ijo.cn/gjykcn/article/abstract/201911029)[60] | 0 | 0 | 0 | 0 | 0 | 0 | 0 | 0 | 0 | 0 | 0 | 0 | 0 | 1 | 0 | 0 | 0 | 0 | 0 |
| [Cui 2017](https://kns.cnki.net/kcms2/article/abstract?v=kMpVSI0yL5e_7Uc1GO8ZLCZWdudZsasdNsWJ3FBm_KHZmPQShSNsYdZf8izrbSXqLvW6cabKl-Cqp0F94RoAPnclMnjAUGfWoHAHyPQ1zZvkLddN3-4ENfh3jCdJmphwhBf-yPzvPZ_WAENyiD8LEg==&uniplatform=NZKPT)[61] | 0 | 0 | 0 | 0 | 0 | 0 | 0 | 0 | 0 | 0 | 0 | 0 | 0 | 0 | 0 | 0 | 0 | 1 | 0 |
| [Zheng et al. 2020](https://d.wanfangdata.com.cn/periodical/ChlQZXJpb2RpY2FsQ0hJTmV3UzIwMjMwODMxEg9sY3lrenoyMDIwMDYwMTQaCGVmNWFseDVw)[62] | 0 | 0 | 0 | 0 | 0 | 0 | 0 | 0 | 0 | 0 | 0 | 0 | 0 | 0 | 0 | 0 | 0 | 0 | 1 |
| [Rong et al. 2020](https://med.wanfangdata.com.cn/Paper/Detail?id=PeriodicalPaper_ykyj202006013&dbid=WF_QK)[63] | 0 | 0 | 0 | 0 | 0 | 0 | 0 | 0 | 0 | 0 | 0 | 0 | 0 | 0 | 0 | 0 | 0 | 0 | 1 |
| [Meng 2020](https://med.wanfangdata.com.cn/Paper/Detail?id=PeriodicalPaper_hgzy202001032&dbid=WF_QK)[64] | 0 | 0 | 0 | 0 | 0 | 0 | 0 | 0 | 0 | 0 | 0 | 0 | 0 | 0 | 0 | 0 | 0 | 0 | 1 |
| [Shen et al. 2020](https://kns.cnki.net/KXReader/Detail?invoice=wBIoEKlszeHtcFy%2F0Z%2BREOZKC7z8jZrn4tLisdjS18NpBvIKKmj5CGpVQ5%2BUgBLXXDxDuWWNpVqsBO05AO6RbwTAQHTY%2B%2BSba%2BUUbstBia6RHMd8cESWjDVwsHNPA%2BnPy5Q2Etid2C%2BL2U1q62YnwYTVc8p1fjt%2FQfeN%2FDyBeR4%3D&DBCODE=CJFD&FileName=SXYY202009033&TABLEName=cjfdlast2020&nonce=7DCBCDE80DCE4964BE1AC9288A44D63E&TIMESTAMP=1697618145455&uid=)[65] | 0 | 0 | 0 | 0 | 0 | 0 | 0 | 0 | 0 | 0 | 0 | 0 | 0 | 0 | 0 | 0 | 0 | 1 | 1 |
| [Liu et al. 2020](https://kns.cnki.net/KXReader/Detail?invoice=cL17%2FeuvhICx%2B3wStNtTOs08nGyRtctDtFgrtU5BrVYv7BVDKP5DNrGyqP3jdnwtODVJTqcVc5iLMFOtPBKL5HpaEbzUtsoQ8ywtRycjli2FyIb3ip1%2BvSsTZ0WeP7zmEnqktP4gdUf6iLaXiAgekgdHhb3igkfPKDcbicGcYAg%3D&DBCODE=CJFQ&FileName=SXYY202005042&TABLEName=cjfdlast2020&nonce=D50B47BFA4E44CB09C71B48484425474&TIMESTAMP=1697619819578&uid=)[66] | 0 | 0 | 0 | 0 | 0 | 0 | 0 | 0 | 0 | 0 | 0 | 0 | 0 | 0 | 0 | 0 | 0 | 0 | 1 |
| [Li et al. 2018](https://kns.cnki.net/KXReader/Detail?invoice=nloUKcS1BQG1dRl0ltHzEt0ZdUbcuPb9wKxqL44WJQNYHKSyhNB8oAr1wbzKLADZgaVMI7mYzHlvpkT3HjVo%2FddP8N4M5RjH6fy96Lg%2FXGNOYcHZB3QnrBJbgfB%2Fp2GDqZ9zI7fDj9ZZHXVz08ai8qxLYL1K1%2BdcORgervnTXoo%3D&DBCODE=CJFD&FileName=XIWS201803035&TABLEName=cjfdlast2018&nonce=081136EDC751497D9344A566D6AAC28D&TIMESTAMP=1697619969060&uid=)[67] | 0 | 0 | 0 | 0 | 0 | 0 | 0 | 0 | 0 | 0 | 0 | 0 | 0 | 0 | 0 | 0 | 0 | 1 | 0 |
| [Zhu et al. 2019](https://d.wanfangdata.com.cn/periodical/ChlQZXJpb2RpY2FsQ0hJTmV3UzIwMjMwODMxEg96amxjeXgyMDE5MDYwMzIaCGpocWdiZ213)[68] | 0 | 0 | 0 | 0 | 0 | 0 | 0 | 0 | 0 | 0 | 0 | 0 | 0 | 0 | 0 | 0 | 0 | 1 | 0 |

1 means that this clinical research was included in the systematic review/meta-analysis; 0 means that this clinical wasn’t included in the SR/MA.

**Table S5**. Methodological quality of included studies based on AMSTAR2 criteria.

| **Study, year** | **Q1** | **Q2** | **Q3** | **Q4** | **Q5** | **Q6** | **Q7** | **Q8** | **Q9** | **Q10** | **Q11** | **Q12** | **Q13** | **Q14** | **Q15** | **Q16** | **Overall quality** |
| --- | --- | --- | --- | --- | --- | --- | --- | --- | --- | --- | --- | --- | --- | --- | --- | --- | --- |
| Song et al, 2011 | Y | PY | N | Y | Y | Y | Y | Y | N | N | Y | N | N | Y | Y | Y | Critically Low |
| Li et al, 2014 | Y | PY | N | Y | N | Y | Y | Y | PY | N | Y | N | N | Y | N | Y | Critically Low |
| Chen et al, 2021 | Y | Y | N | Y | N | Y | Y | Y | Y | N | Y | Y | Y | Y | Y | Y | High |
| Gan et al, 2022 | Y | PY | N | Y | N | Y | Y | Y | PY | N | Y | Y | Y | Y | Y | Y | Moderate |
| Gong et al, 2017 | Y | PY | N | Y | Y | Y | Y | Y | Y | N | Y | N | N | Y | Y | Y | Low |
| Lanca et al, 2023 | Y | PY | N | Y | N | N | Y | Y | Y | N | Y | N | N | Y | Y | Y | Low |
| Tran et al, 2021 | Y | PY | N | PY | Y | Y | Y | Y | Y | N | Y | N | N | Y | N | Y | Critically Low |
| Yang et al, 2022 | Y | PY | N | PY | Y | Y | Y | Y | Y | N | Y | N | N | Y | Y | Y | Low |
| Zhao et al, 2020 | Y | PY | N | Y | Y | Y | Y | PY | Y | N | Y | Y | Y | Y | Y | Y | Moderate |
| Zheng et al, 2022 | Y | PY | N | Y | Y | N | Y | Y | Y | N | Y | N | N | Y | Y | Y | Low |
| Wei et al, 2023 | Y | PY | Y | Y | N | Y | Y | Y | Y | N | Y | N | N | Y | Y | Y | Low |
| Wang et al, 2020 | Y | PY | N | Y | Y | Y | Y | Y | Y | N | Y | Y | Y | Y | N | Y | Low |
| Wang et al, 2023 | Y | PY | N | PY | N | Y | Y | Y | Y | N | Y | Y | Y | N | Y | Y | Moderate |
| Tsai et al, 2021 | Y | Y | N | Y | N | Y | Y | Y | Y | N | Y | Y | Y | Y | N | Y | Low |
| Sun et al, 2022 | Y | PY | N | Y | N | Y | N | Y | PY | N | Y | N | N | Y | Y | Y | Critically Low |
| [Kumari et al, 2022](https://pubmed.ncbi.nlm.nih.gov/?term=Kumari+S&cauthor_id=36993096) | Y | PY | N | PY | Y | Y | Y | Y | PY | N | Y | N | N | N | Y | Y | Moderate |
| Zhang et al, 2023 | Y | PY | N | Y | Y | Y | PY | N | PY | N | Y | N | N | Y | Y | N | Moderate |
| Fan et al, 2021 | Y | PY | N | PY | Y | Y | PY | Y | Y | N | Y | N | Y | Y | Y | Y | Moderate |
| Gao et al, 2021 | Y | PY | N | PY | N | Y | Y | Y | Y | N | Y | N | Y | Y | Y | Y | Moderate |

Q1: Were patient/problem, intervention, comparsion and outcome (PICO) components taken into consideration when drawing up the research questions and inclusion criteria? Q2: Does the review report explicitly state that the methods of the review were decided before commencing the review, and if these methods were changed does the report justify the change? Q3: Have the authors provided an explanation for their choice of the study designs they include in the review? Q4: Was the authors’ strategy for literature search sufficiently comprehensive? Q5: Were any duplicates of studies selected? Q6: Were any duplicate data extracted? Q7: Were excluded studies listed and a justification given for the exclusion? Q8: Is the detail with which the authors describe the studies adequate to the task? Q9: Has the risk of bias in individual studies been addressed satisfactorily through an appropriate technique? Q10: Are the individual studies’ funding sources reported in the review? Q11: Have the authors of the review employed appropriate methods to statistically combine the results of any meta-analysis conducted? Q12: Have the review authors assessed what impact risk of bias in individual studies might have on the results of their meta-analyses or on other evidence formation? Q13: Is risk of bias in the individual studies adequately addressed by the authors in the discussion and interpretation of their review’s results? Q14: Where heterogeneity was observed in the review results, have the authors attempted a satisfactory discussion and explanation thereof? Q15: If quantitative synthesis was performed, have the authors adequately addressed the issue of publication bias (small study bias) and offered a discussion of how it might be likely to impact the review results? Q16: Have the review authors reported on any conflicts of interest, including through funding of their review, which might arise? AMSTAR2, Assessment of Multiple Systematic Reviews 2; N, no: negative response or response not available; PY, partial yes: incomplete adherence to the criteria; X, no meta-analyses performed; Y, yes: positive response.

AMSTAR 2 Critical domains: Protocol registered before commencement of the review(item 2); Adequacy of the literature search(item 4); Justification of excluding individual studies(item 7); Risk of bias from individual studies being included in the review(item 9); Appropriateness of meta-analytical methods(item 11); Consideration of risk of bias when interpreting the results of the review(item 13); Assessment of presence and likely impact of publication bias(item 15).
